# Supplementary figures and images for: The impact of sleep on breast cancer-specific mortality: a Mendelian randomisation study
Source: BMC Cancer. 2025 Feb 26;25:357. doi: 10.1186/s12885-025-13681-4 (PMC11863467; doi:10.1186/s12885-025-13681-4)

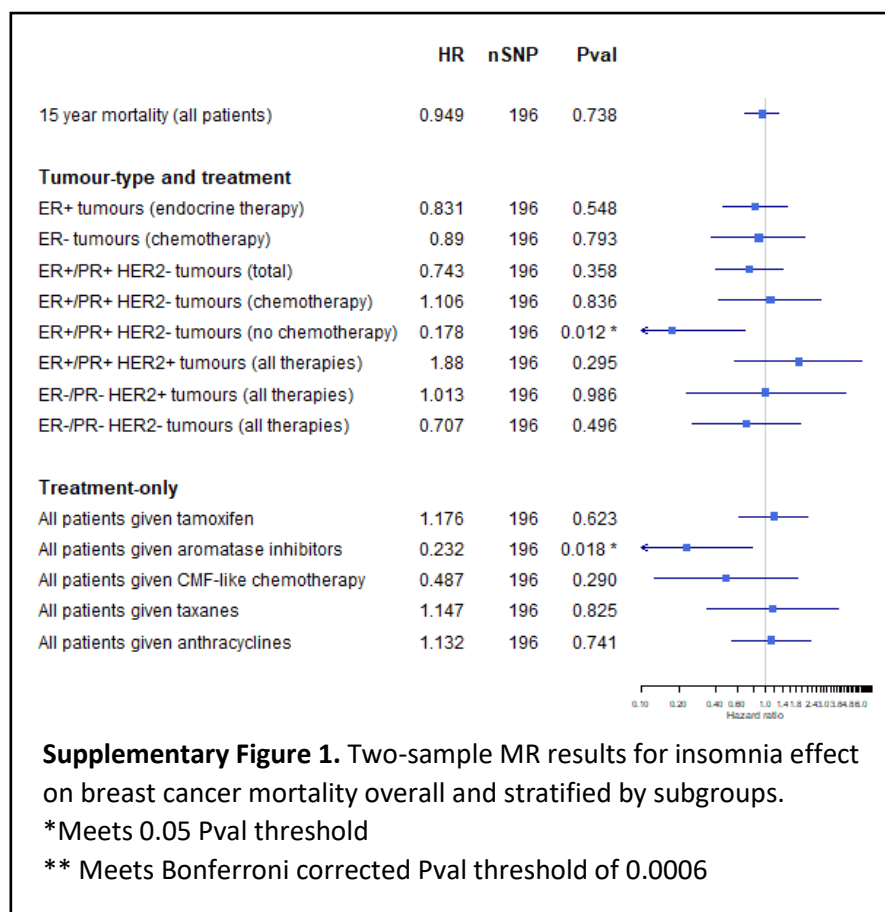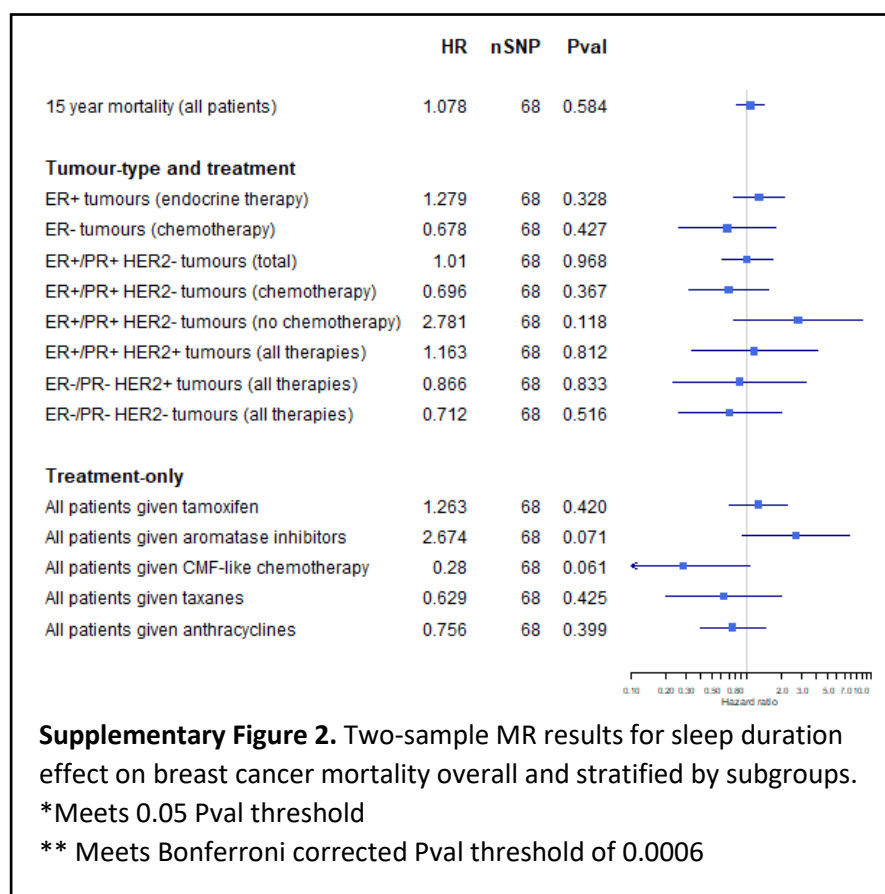

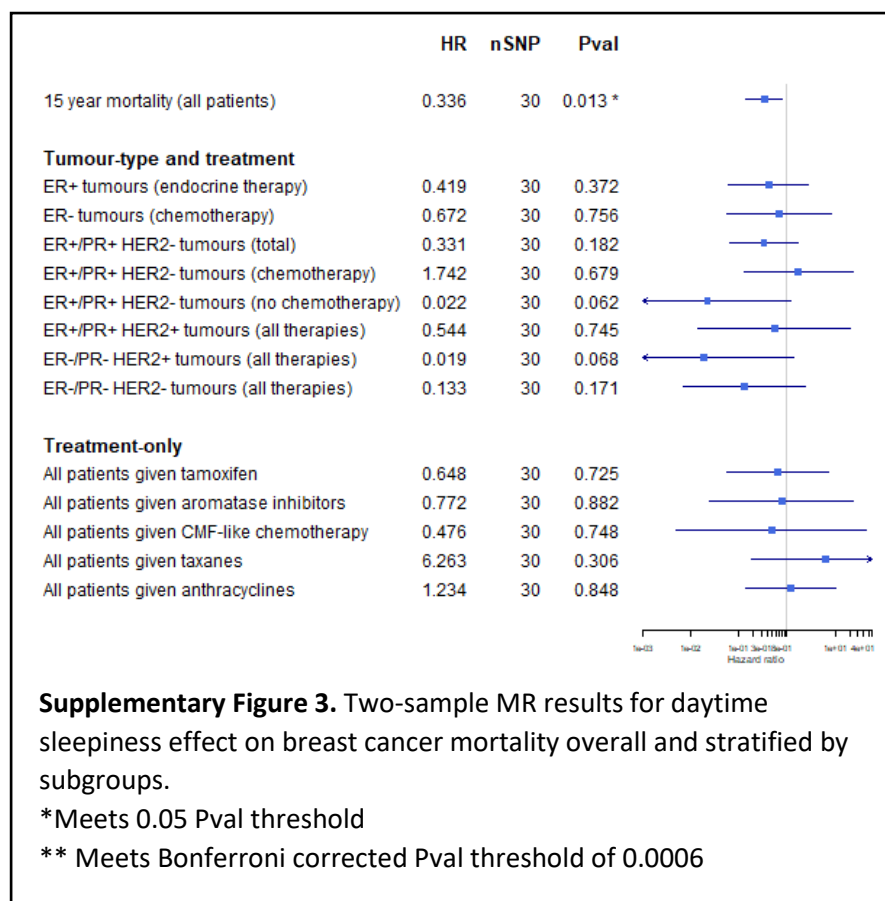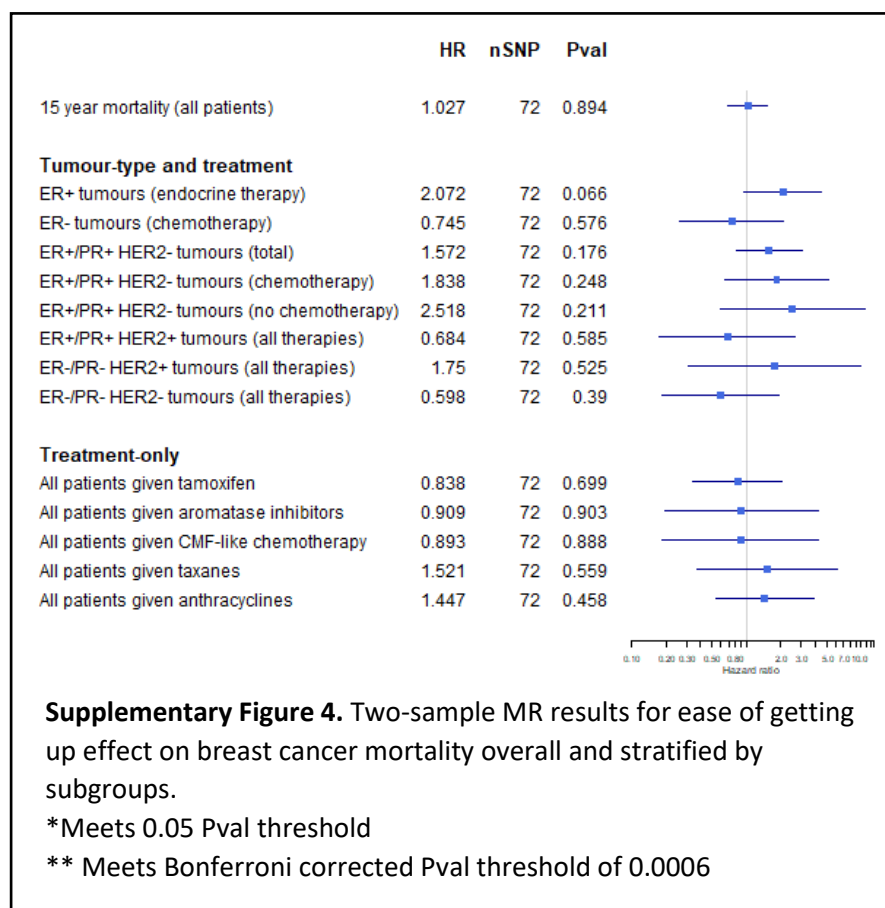

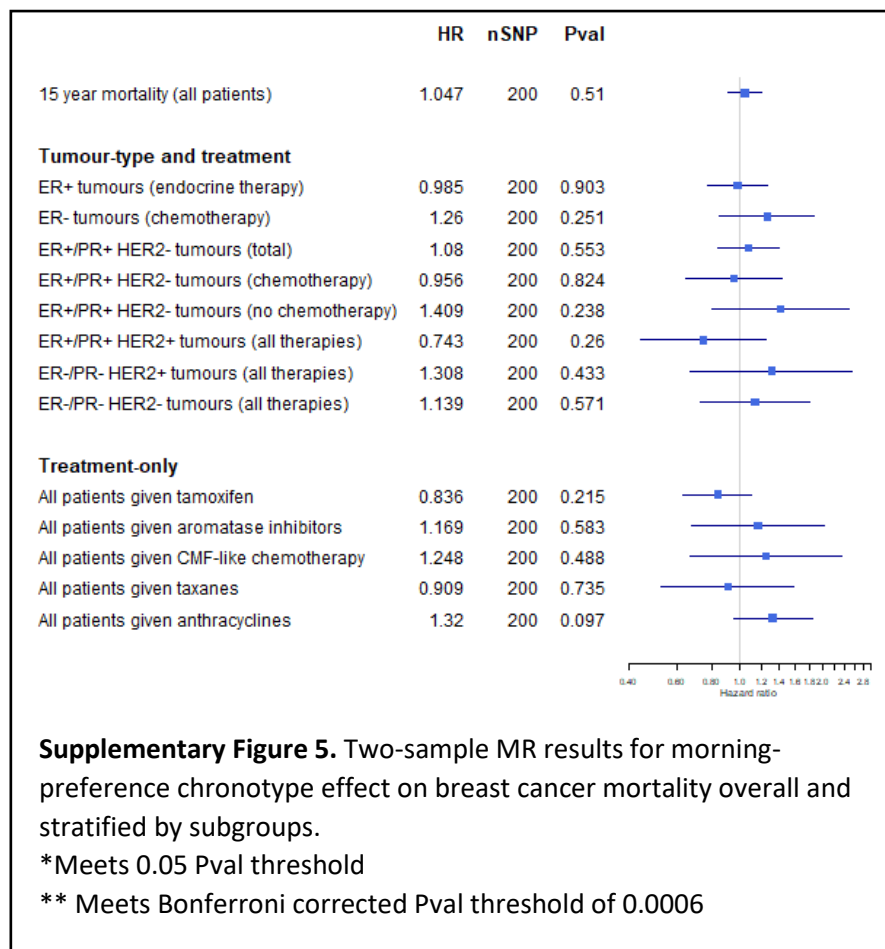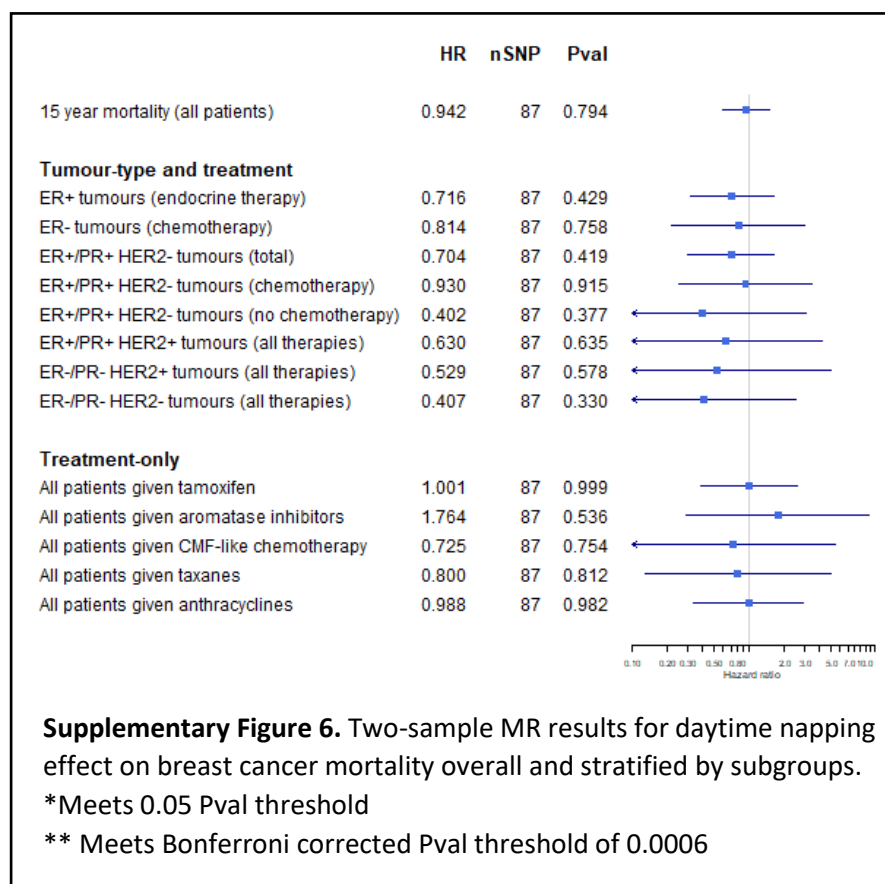

Supplement: Supplementary file 3 — Supplemenatry Material 3. [file 12885_2025_13681_MOESM3_ESM.pdf]
